# Endothelial dysfunction is associated with carotid plaque: a cross-sectional study from the population based Northern Manhattan Study

**DOI:** 10.1186/1471-2261-6-35

**Published:** 2006-08-17

**Authors:** Tatjana Rundek, Rameet Hundle, Elizabeth Ratchford, Romel Ramas, Robert Sciacca, Marco R Di Tullio, Bernadette Boden-Albala, Yumiko Miyake, Mitchell SV Elkind, Ralph L Sacco, Shunichi Homma

**Affiliations:** 1 Department of Neurology, College of Physicians and Surgeons, Columbia University, New York, US; 2 Department of Medicine, College of Physicians and Surgeons, Columbia University, New York, US; 3 Gertrude H. Sergievsky Center, College of Physicians and Surgeons, Columbia University, New York, US; 4 Division of Sociomedical Sciences, Joseph P. Mailman School of Public Health, Columbia University, New York, US; 5 Department of Epidemiology, Joseph P. Mailman School of Public Health, Columbia University, New York, USA

## Abstract

**Background:**

Impaired vascular function occurs early in atherogenesis. Brachial flow mediated dilatation (FMD) is a non-invasive measure of vascular function and may be an important marker of preclinical atherosclerosis. Data on the association between FMD and carotid plaque in multi-ethnic populations are limited. The objective of this study was to determine whether endothelial dysfunction is independently associated with carotid plaque in a community of northern Manhattan.

**Methods:**

In the population-based Northern Manhattan Study (NOMAS), high-resolution B-mode ultrasound images of the brachial and carotid arteries were obtained in 643 stroke-free subjects (mean age 66 years; 55% women; 65% Caribbean-Hispanic, 17% African-American, 16% Caucasian). Brachial FMD was measured during reactive hyperemia. Maximum carotid plaque thickness (MCPT) was measured at the peak plaque prominence.

**Results:**

The mean brachial FMD was 5.78 ± 3.83 %. Carotid plaque was present in 339 (53%) subjects. The mean MCPT was 1.68 ± 0.82 mm, and the 75^th ^percentile was 2.0 mm. Reduced FMD was significantly associated with increased MCPT. After adjusting for demographics, vascular risk factors, and education, each percent of FMD decrease was associated with a significant 0.02 mm increase in MCPT (p = 0.028). In a dichotomous adjusted model, blunted FMD was associated with an increased risk of MCPT ≥ 2.0 mm (OR, 1.11 for every 1% decrease in FMD; 95% CI, 1.03–1.19).

**Conclusion:**

Decreased brachial FMD is independently associated with carotid plaque. Non-invasive evaluation of endothelial dysfunction may be a useful marker of preclinical atherosclerosis and help to individualize cardiovascular risk assessment beyond traditional risk factors.

## Background

Atherosclerosis is characterized by a low-grade systemic inflammatory response and endothelial dysfunction [1]. Endothelial dysfunction is comprised of increased expression of cellular adhesion molecules, loss of anticoagulant properties, and increased vascular tone due to loss of bioavailability of vasodilatory endothelial nitric oxide [2]. Flow-mediated dilatation (FMD) of the brachial artery is a reliable and reproducible non-invasive tool used to evaluate endothelial function [3]. Impaired brachial FMD has been associated with traditional cardiovascular risk factors and has been observed to predict cardiovascular events in selected high-risk groups of patients [4,6].

Carotid plaque and intima-media thickness (IMT) detected by non-invasive high-resolution ultrasound are markers of systemic subclinical atherosclerosis and strong predictors of future myocardial infarction (MI) and stroke [7,9]. Several case-control studies have observed an association between decreased brachial FMD and increased IMT, thus linking endothelial dysfunction to atherogenesis [10]. However, these studies were limited by their small sample size and by issues in study design. Recently, no significant correlation between brachial FMD and carotid IMT was observed among middle-aged healthy men [11]. In the same population, FMD was not associated with traditional vascular risk factors; while the opposite was reported in individuals at low cardiovascular risk in a recent meta-analysis [12] as well as in other cohorts [13]. There is an ongoing debate whether FMD is associated with other imaging markers of systemic subclinical atherosclerosis and to which extent is modified by the effects of vascular risk factors. Carotid plaque may be a distinctive phenotype of atherosclerosis and not a continuum of increased wall thickness or IMT progression [10,14]. In this study, we sought to examine the association between endothelial function and carotid plaque in an urban population-based multiethnic cohort, and to investigate possible interactions with age, race-ethnicity, and traditional vascular risk factors.

## Methods

### Study population

The study population was obtained from the Northern Manhattan Study (NOMAS), an ongoing community-based study designed to investigate vascular risk factors and the incidence of vascular events, to identify novel risk factors, and to provide data on the association of various vascular risk factors and subclinical atherosclerosis in different race-ethnic groups in northern Manhattan [15,16]. NOMAS is comprised of 3,298 adult subjects without prior MI or stroke. After several months of piloting brachial FMD in our cohort and working out the technical methods, we began collecting data on FMD in 1998. Participants who were undergoing cardiac echo were selected to undergo brachial FMD testing. Between January 1998 and April 2001, we obtained high quality FMD images among 842 subjects of whom 643 had both carotid ultrasonography and brachial FMD measurements performed. These subjects were not significantly different from the parent cohort with respect to demographic and risk factors. The cardiovascular risk factors were collected using the CDC Risk Factor Surveillance Instrument. Hypertension was defined as a systolic blood pressure ≥ 140 mmHg or a diastolic blood pressure ≥ 90 mmHg, or the patient's self-report of a history of hypertension or antihypertensive medication use. Diabetes mellitus was defined as either a fasting blood glucose level ≥ 126 mg/dL or the subject's self-report of diabetes, insulin use, or oral hypoglycemic use. Hypercholesterolemia was defined as a history of elevated cholesterol, taking medications for elevated cholesterol or a total cholesterol level > 240. Current tobacco use was defined as smoking within the past year. Alcohol use was defined as more than one drink per month. Physical activity was categorized as any, if any participation in usual leisure physical activities was reported, or none. The study was approved by the Columbia University Medical Center Institutional Review Board, and all patients gave written informed consent.

### Assessment of endothelial function

Endothelial function was assessed by measuring brachial artery FMD in response to reactive hyperemia using high-resolution B-mode ultrasound [3]. Participants fasted for 12 hours prior to the brachial FMD examination. They were asked not to ingest substances that might affect flow mediated dilatation (FMD) such as caffeine, or use tobacco at least 6 hours prior to the appointment, and to avoid vasoactive medications for 24 hours prior to the study. The brachial artery diameter was measured 6 cm proximal to the antecubital crease using a 7–15 MHz linear array transducer (Philips 5500, Andover, MA). Flow-mediated dilatation was measured as the response to reactive hyperemia induced by a 5-minute blood pressure cuff occlusion of the upper arm. Inflation pressure was 200 mmHg if the subject's pre-examination systolic blood pressure was less than 150 mmHg, or 50 mmHg above the systolic blood pressure if it was greater than 150 mmHg but less than 180 mmHg. End-diastolic images were acquired at baseline and 1 minute after cuff deflation. Data was analyzed using CVI analysis and acquisition software (Data Translation, MA). Flow-mediated dilatation was expressed as FMD = [(brachial artery diameter at peak hyperemia - diameter at rest)/diameter at rest × 100]. Intra-observer and inter-observer variability for FMD measurements were 1.3% and 2.7%, respectively (n = 15).

### Assessment of carotid plaque

Carotid artery plaque was assessed according to a standard scanning and reading protocols [17] using high-resolution B-mode ultrasound GE LOGIQ 700 system with a multifrequency 9–13 MHz linear-array transducer. The internal carotid and common carotid arteries as well as the bifurcations were examined for the presence of atherosclerotic plaque, defined as an area of focal wall thickening or protrusion into the lumen at least 50% greater than surrounding total wall thickness. Maximum carotid plaque thickness (MCPT) was measured at the peak plaque prominence from any of the three carotid artery segments using a semi-automatic IMAGE-Pro V.5.2 software (Microsoft). If plaque was not identified, plaque thickness was recorded as zero. The intraclass correlation coefficients for within and between reader variability of MCPT measurements were 0.94 and 0.77, respectively (n = 88) [18].

### Statistical analysis

All statistical analyses were performed using SAS 8.2 (SAS Institute, Cary, NC). All continuous data are presented as a mean value ± SD, and all categorical data are reported as a percentage or absolute number. Student's *t *tests and Chi-square tests were used to assess differences between groups. The association between FMD and MCPT was analyzed by multivariable linear and logistic regression analyses. The dependent variable, MCPT, was analyzed as a continuous variable, and dichotomized using the 75^th ^percentile cut off level of the plaque thickness distribution. These analyses were adjusted for demographics, hypertension, hypercholesterolemia, diabetes, education, physical activity, alcohol, and current smoking. The results are reported as slopes (β) for the linear regression models and as odds ratios (OR) for the logistic regression. To test for differences in the effect of FMD on MCPT among age and race-ethnic groups, interaction terms were added to the multivariate models.

## Results

### Study population

The study population included 643 subjects with a mean age of 66.1 ± 8.5 years (range, 54–94 years), 55% female, 65% Hispanic, 17% African American, and 16% Caucasian (Table 1). The prevalence of risk factors was as follows: 67% hypertension, 24% diabetes, 50% hypercholesterolemia, 43% subjects reported consuming more than one glass of alcohol per month, 13% were current smokers, 49% were physically inactive, and 41% completed high school. The mean brachial artery diameter at baseline was 3.9 ± 0.7 mm, and the mean absolute change was 0.22 ± 0.14 mm, with a mean percent change in diameter (FMD) of 5.78 ± 3.83. Significant correlation was found between FMD and baseline brachial artery diameter (r = -.414, p<0.001) was found. Carotid plaque was present in 339 subjects (53%). The mean MCPT was 1.68 ± 0.82 mm (range 0 – 8.0 mm). The 75^th ^percentile of the MCPT distribution was 2.0 mm.

**Table 1:** Characteristics of study subjects from the Northern Manhattan Study

	**N**	**Prevalence (%) or Mean ± SD**
Total	643	--
Age	643	66.1 ± 8.5 years
Men	290	45 %
Hispanic	417	65 %
African American	109	17 %
Caucasian	102	16 %

Hypertension	431	67 %
Diabetes mellitus	154	24 %
Hypercholesterolemia	322	50 %
Alcohol consumption	276	43 %
Current smoking	84	13 %
Physical inactivity	315	49 %
Completed high school	264	41 %

Carotid plaque	339	53%
MCPT (mm)	643	1.68 ± 0.82
75^th ^percentile		2.00
Baseline diameter of the brachial artery (mm)	643	3.90 ± 0.71
FMD (%)	643	5.78 ± 3.83

FMD = flow mediated dilatation, MCPT= maximum carotid plaque thickness

### Relationship between brachial FMD and carotid plaque (MCPT)

**Figure 1:**
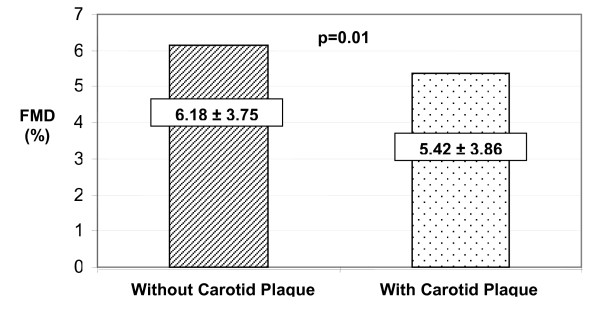
The mean ( ± SD) values of brachial FMD among 304 (47%) subjects without carotid plaque and among 339 (53%) subjects with carotid plaque.

Mean FMD was lower among subjects with carotid plaque (5.42 ± 3.86) than among subjects without plaque (6.18 ± 3.75), p = 0.01 (Figure 1). Subjects in the lowest quartile of FMD (<2.81) were more likely to have MCPT ≥ 2.0 mm, with 22% of them compared to 9% in subjects in the highest quartile of FMD (≥ 8.38), p<0.01 (data not shown).

Reduced FMD was significantly associated with increased MCPT in a univariate regression model (β = -0.038, p<0.001). After adjusting for demographics and risk factors, and baseline diameter of the brachial artery the magnitude of the effect was reduced but FMD remained significantly associated with increased MCPT (Table 2, β = -0.021, p = 0.027). No significant interactions between FMD and hypertension, diabetes, or smoking were found.

**Table 2:** Association between brachial FMD and maximum carotid artery thickness (MCPT)

	**β **	**p**
**FMD **	**-0.021**	**0.027**
**Age (per year)**	**0.041**	**<0.001**
**Current Smoking**	**0.378**	**0.001**
Male	0.073	0.366
Systolic Blood Pressure (per mm Hg)	0.001	0.566
Diabetes	0.098	0.292
High Cholesterol	0.140	0.060
Completed high school	0.069	0.363
Physical Activity	0.038	0.650
Alcohol Consumption	-0.019	0.899
Baseline diameter of the brachial artery (mm)	0.193	0.072

In a dichotomous model, lower FMD was significantly associated with MCPT ≥ 2.0 mm (OR, 1.10 for every 1% decrease in FMD; 95% CI, 1.04–1.18) in a univariate and in a multivariate model (Table 3).

**Table 3:** Association between brachial FMD and MCPT ≥ 2.0 mm

	Odds Ratio	**95% **Confidence Interval
**FMD (per 1% decrease)**	**1.10**	**1.04 – 1.18**
**Age (per year)**	**1.11**	**1.08 – 1.11**
**Current Smoking**	**2.30**	**1.28 – 4.44**
Male	1.11	0.69 – 1.92
Systolic Blood Pressure (per mmHg)	0.99	0.97 – 1.01
Diabetes	1.39	0.85 – 2.66
High Cholesterol	1.44	0.87 – 2.41
Completed high school	1.04	0.62 – 1.69
Physical Activity	0.88	0.57 – 1.41
Alcohol Consumption	1.05	0.67 – 1.77
Baseline diameter of the brachial artery (mm)	0.87	0.71 – 1.84

### Interactions with age, sex, and race-ethnicity

No significant differences were detected in the effect of FMD on MCPT among various race-ethnic groups in either the univariate (p = 0.37) or multivariable (p = 0.27) logistic regression models although the OR for Caucasians (1.23; 95% CI, 0.97–1.49) showed a somewhat larger effect than for African Americans (1.04; 95% CI, 0.89–1.20) or Caribbean Hispanics (1.06; 95% CI, 0.97–1.16). The differences were found for the FMD effect among those aged 65 or older (adjusted OR = 1.16; 95% CI, 1.03–1.49), than in those younger than 65 years (adjusted OR = 1.01; 95% CI, 0.90–1.14), although not statistically significant (p = 0.06). No significant difference in the association of FMD with MCPT was found between men and women.

## Discussion

We found a significant association between endothelial dysfunction and presence of carotid plaque in a population-based cohort. Impairment of brachial FMD was more pronounced among those with carotid plaques thicker than 2 mm. This thickness was associated with a high cardiovascular risk [7,9]. The inverse relationship between FMD and carotid plaque was independent of demographics and traditional vascular risk factors, raising the possibility that endothelial dysfunction may directly promote atherogenesis. Our observation emphasizes the importance of evaluating endothelial function in the assessment of vascular risk beyond traditional risk factors. Furthermore, carotid artery plaque assessment in the combination with FMD may be a useful monitoring tool for high risk individuals.

Other investigators have assessed the relationship between FMD and subclinical atherosclerosis. AA mong 2,109 young healthy adults from the Young Finns study (ages 24 to 39), brachial FMD was inversely and significantly associated with carotid IMT [19]. In contrast, no significant correlation between brachial FMD and carotid IMT was observed among 1,578 middle-aged healthy men from the Firefighters and their Endothelium (FATE) study [11]. Although the FATE study population consisted of only men who were slightly older than the Young Finns population (mean age of 49 ± 10 years), the discrepancy in the results from these two population is striking. A different IMT measurement technique is the most evident dissimilarity. In the Young Finns study, IMT was measured only from the far wall of the distal part of the common carotid artery; while a more complex carotid IMT score including the internal and common carotid arteries and bifurcation was used in the FATE study. The results from the FATE study are somehow contraintuitive as a more complex and presumably more detailed analysis of IMT was less likely to show a significant relationship with FMD. Furthermore, even if one assumes differences in IMT measurement techniques to be responsible for these discrepant results, it cannot explain almost total lack of association between FMD and conventional cardiovascular risk factors in the FATE study. Other characteristics of the study populations may also have accounted for the conflicting results. None of the studies reported on the presence of carotid plaque.

In our study, carotid plaque thickness, not carotid IMT, was used as a measure of subclinical atherosclerosis, and was significantly associated with reduced FMD. Carotid plaque may be a distinctive phenotype of atherosclerosis and not just a continuum of increased wall thickness or IMT progression. Carotid IMT, especially in early development, represents mainly hypertensive hypertrophy of the media and is under substantial genetic control [13]. A considerable heritability of carotid IMT of 40% was recently reported [20]; while the heritability of carotid plaque was insignificant. Indeed, carotid plaque is strongly influenced by the environmental factors including a control of lipids, monocytes, smooth muscle cells, and oxidative stress. Therefore, endothelial dysfunction may be more likely associated with plaque due to environmental exposures and less likely associated to IMT which is under significant genetic control. Several studies have provided evidence that carotid plaque predicts prevalent CAD better than does IMT [14,21]. FMD and carotid plaque may also be independent predictors of vascular outcome [22]. Reduced availability of endothelium-derived nitric oxide, as reflected by reduced FMD, may modulate the impact of atherosclerotic plaque by enhancing its progression and rapidly increasing the risk for vascular events [23]. Thus, the functional and structural assessments of the arterial wall may be fundamental determinants in the prognosis of vascular disease. Nonetheless, relatively small and limited case-control studies and case series have examined the association between FMD and carotid plaque [10].

The strength of association between FMD and carotid plaque in our study was similar among men and women, and various race-ethnic groups. Although preliminary data suggest that FMD differs by sex and race-ethnicity and that these differences may parallel differential cardiovascular risk [24], it remains to be determined whether sex and racial differences in endothelial function explain the difference in CVD morbidity and mortality. In our cohort, the association between FMD and carotid plaque was more pronounced among subjects older than 65 years. It has already been recognized that many structural and vascular changes occur with aging even among subjects without overt CVD [25].

Numerous studies have reported on the associations between FMD and vascular risk factors. We have previously observed the associations of FMD with diabetes and hypertension [13]. However, we did not find any interactions between FMD and traditional risk factors on the risk of carotid plaque. In contrast, in the recent meta-analysis, FMD was related to the risk factors only in the low risk populations [12]. This discrepancy can be explained by the different demographics of the studied populations. In addition the biology of endothelium where its dysfunction is an initial step in atherogenesis after which an addition of risk factors may not cause a further decline of endothelial function. In meta-analysis however, other confounders including variations between and within the reported studies may have influenced the results. Smoking may be an important confounder modifying endothelial dysfunction [26]. We did not observe a significant interaction between smoking and FMD. Our observation contributes to the hypothesis that endothelial dysfunction may directly lead to atherogenesis, although some residual confounding cannot be entirely excluded. The mechanism by which endothelial dysfunction may promote plaque formation independently of other risk factors is not clear. Imbalance in the production and degradation of nitric oxide within the arterial wall may play a major role by directly influencing atherogenesis through leukocyte adhesion, lipid deposition, vascular smooth muscle cell growth, and thrombosis [2].

Strengths of our study include the population-based design, the diverse ethnic population, and ongoing follow-up, which will allow for the prospective evaluation of FMD as a clinically useful measure of vascular risk. Our study also has several limitations. For any such study, temperature, foods, medications, and sympathetic stimuli may all affect FMD. Attempts were made to control these factors to the greatest extent possible during subject preparation, yet these factors depend on patient cooperation, and any aberration may have influenced FMD results. Measurements of carotid IMT were not available in this group of subjects therefore the associations between FMD and IMT were not assessed. However, carotid plaque is an accepted surrogate marker of atherosclerosis and may be even a stronger predictor of vascular events than IMT [14]. The reported associations are cross-sectional, not allowing for determination of whether FMD changes precede plaque formation.

## Conclusion

Endothelial dysfunction in this multi-ethnic population of older men and women is associated with carotid plaque. Carotid plaque is a recognized measure of subclinical atherosclerosis and a marker of future cardiovascular events. Impaired FMD predicts vascular events in selected high-risk groups of patients, and several large ongoing population-based studies, including MESA [27], CHS [7], the Framingham Heart Study [4], and NOMAS [16] will determine whether brachial FMD independently identifies individuals at risk for developing cardiovascular disease in the general population.

## Competing interests

The author(s) declare that they have no competing interests.

## Authors' contributions

TR conceived of the study, carried out carotid ultrasound studies, participated in the data analysis and interpretation and drafted the manuscript. RH participated in the design of the study, interpretation of data, and drafting of the manuscript. ER participated in the conception and the study design, interpretation of data, and drafting of the manuscript. RR carried out carotid ultrasound studies, participated in the interpretation of ultrasound data, and participated in the critical review of the manuscript. YM participated in interpretation of the results, analysis of the ultrasound data and in critical review of the manuscript. RS participated in the conception and the design of the study, performed the statistical analysis and interpretation and in critical review of the manuscript. MRDT participated in the coordination of the study, analysis and interpretation of data and in critical review of the manuscript. BB-A participated in the study coordination, data analysis and interpretation and in critical review of the manuscript. MSVE participated in the data analysis and interpretation and critically reviewed the manuscript. RLS participated in the study design, data analysis and interpretation, and critically reviewed the manuscript. SH participated in the study design, data analysis and interpretation, and critically reviewed the manuscript. All authors read and approved the final manuscript.

## Pre-publication history

The pre-publication history for this paper can be accessed here:


